# Very low likelihood that cultivated oysters are a vehicle for SARS-CoV-2: 2021–2022 seasonal survey at supermarkets in Kyoto, Japan

**DOI:** 10.1016/j.heliyon.2022.e10864

**Published:** 2022-10-06

**Authors:** Yasuko Yamazaki, Uraiwan Thongchankaew-Seo, Wataru Yamazaki

**Affiliations:** a Center for Southeast Asian Studies, Kyoto University, 46 Shimoadachicho, Yoshida, Sakyo-ku, Kyoto 606-8501, Japan; b Kyoto University School of Public Health, Konoe-cho, Yoshida, Sakyo-ku, Kyoto 606-8303, Japan

**Keywords:** SARS-CoV-2, Cultivated oysters, Quantitative RT-PCR, Survey

## Abstract

The pandemic caused by novel coronavirus disease of 2019 (COVID-19) is a global threat. Wastewater surveillance in Japan and abroad has led to the detection of SARS-CoV-2, causing concern that SARS-CoV-2 in the feces of infected persons may contaminate the aquatic environment. Bivalves such as oysters cultivated in coastal areas are known to filter and concentrate viruses such as norovirus present in seawater in their bodies; however, whether they do so with SARS-CoV-2 is unknown. Therefore, we examined cultivated oysters sold in Japan for the presence of SARS-CoV-2 between October 2021 and April 2022 to clarify the extent of viral contamination and evaluate the risk of food-borne transmission of SARS-CoV-2. Porcine epidemic diarrhea virus (PEDV), known as pig coronavirus, was used to spike midgut-gland samples as a whole process control. The presence of SARS-CoV-2 and PEDV was investigated using a modified polyethylene glycol precipitation method and RT-qPCR. While all samples spiked with the whole process control were positive, no SARS-CoV-2 was detected in any of the 145 raw oyster samples surveyed, despite a marked increase in infections caused by the Omicron variant from January to April 2022 in Japan. Therefore, our results suggest that with well-developed sewage treatment facilities, consumption of oysters cultivated in coastal areas may not be a risk factor for SARS-CoV-2 outbreaks.

## Highlights

• SARS-CoV-2 was not detected in any of the 145 commercial raw oysters in Kyoto, Japan.
• Our modified method successfully detected the spiked virions without inhibition by the midgut component.
• Human transmission of SARS-CoV-2 via commercial oysters in Japan is unlikely.
• Oyster cultivation may not be a risk factor for the spread of SARS-CoV-2 in Japan.

## 1 Introduction

The novel coronavirus disease of 2019 (COVID-19) pandemic caused by severe acute respiratory syndrome coronavirus 2 (SARS-CoV-2) is a global threat (WHO, 2022). The virus can be transmitted directly from an infected person or indirectly via droplets originating from the saliva and oral cavity of an infected person (Jones et al., 2020; Zhang et al., 2020). The virus has also been reported to be present in the feces of infected persons (Ali et al., 2021; Hata et al., 2021; Jones et al., 2020; Kitajima et al., 2020; Tran et al., 2021). Feces containing SARS-CoV-2 enter the sewage system, where they are treated in septic systems before passing into the natural environment, such as rivers and oceans. Wastewater surveillance, which has been conducted in Japan and overseas for early detection of outbreaks, has revealed the presence of SARS-CoV-2 in the waterways (Ali et al., 2021; Hata et al., 2021).

Oysters and other bivalves are known as ‘filter feeders’ because they filter organic matter, including viruses and bacteria, present in seawater through their midgut gland and concentrate them in their bodies (Brnić et al., 2022; Desdouits et al., 2021; Le Guernic et al., 2022; Mancusi et al., 2022; Polo et al., 2021). Previous studies have elucidated a marked infection cycle that arises when norovirus from the feces of infected people seeps into the aquatic environment through sewage and accumulates in the bodies of oysters and other bivalves cultivated in coastal areas.

The latter are consumed by humans without sufficient heating needed to kill the virus (Brnić et al., 2022; Desdouits et al., 2021; Le Guernic et al., 2022). Although several studies have reported a low risk of SARS-CoV-2 transmission through food (Bondad-Reantaso et al., 2020; Godoy et al., 2021), concern remains that a similar infection cycle may arise with SARS-CoV-2, although no details of such a cycle are yet known.

While two countries in Europe have reported detecting SARS-CoV-2 in both natural and cultivated bivalves, there have been no such reports in Asia, including Japan (Mancusi et al., 2022; Polo et al., 2021). Methods for detecting norovirus in oysters, other foods and fecal matter have been reported in Europe (EFSA, 2019; ISO, 2017) and by government testing guidelines in Japan (Imamura et al., 2021; MAFF, 2019; MHLW, 2003; NIHS, 2010). However, no method has been established for detecting SARS-CoV-2 in bivalves, with studies from European nations that reported detecting the virus having only modified the method used for norovirus (Brnić et al., 2022; Desdouits et al., 2021; Le Guernic et al., 2022; Mancusi et al., 2022; Polo et al., 2021). Since both infectious viral particles and non-infectious fragmented RNAs accumulate in the midgut gland of oysters, methods used to detect RNA directly from oyster homogenates may overestimate the actual risk (Imamura et al., 2021, MAFF, 2019, 2021; Polo et al., 2021).

In this study, we developed a more stable method for concentrating and detecting viral particles such as those from SARS-CoV-2 by improving the polyethylene glycol (PEG) precipitation method, the standard method used to detect norovirus in bivalves. We extracted RNA from both PEG precipitates and midgut-gland pellets, to which the virus may be nonspecifically adsorbed, using an automated nucleic acid extractor, before conducting RT-qPCR detection. We used this method to detect SARS-CoV-2 in 145 cultivated oysters commercially sold in 23 supermarkets in Kyoto to elucidate the extent of viral contamination of cultivated oysters and evaluate the risk of SARS-CoV-2 infection via the consumption of oysters in Japan.

## 2 Materials and methods

### 2.1 Number of positive COVID-19 cases

The number of positive COVID-19 cases in Japan between October 2021 and June 2022, the period during which oysters were surveyed in this study, was obtained from the Japanese Ministry of Health, Labour and Welfare (MHLW)’s website (www.mhlw.go.jp/stf/covid-19/open-data.html).

### 2.2 PEG solution

PEG solution was prepared by dissolving 30 g of polyethylene glycol 6000 (Nacalai Tesque, Inc., Kyoto, Japan) and 14.61 g of sodium chloride (Nacalai Tesque) in 100 mL of distilled water. The mixture was stirred and then autoclaved at 121 °C for 15min, and stored at room temperature until use.

### 2.3 Spiking of oyster midgut glands

Cultivated oysters (*Crassostrea gigas*) purchased by mail order in three different lots (from producers in Miyagi, Mie, and Hiroshima) from May to June 2021 were used in the spike test. Prior to the spike test, we confirmed that all three lots of oysters were negative for SARS-CoV-2 using the modified PEG precipitation, automated nucleic acid extraction and real-time RT-PCR methods described below. Once confirmed, known concentrations of SARS-CoV-2 inactivated by heating at 65 °C for 30 min (390,000 copies/μL; ATCC VR1986HK, Lot. 70042082) and porcine epidemic diarrhea virus (PEDV, 10,000,000 copies/μL; NK94-P6), a porcine coronavirus, were added to 10% homogenates of oyster midgut glands. Details of the technique used to prepare the homogenates and the modified PEG precipitation method are provided below.

To monitor changes in viral load, automated nucleic acid extraction and two types of real-time RT-PCR were used to compare changes in the amounts of SARS-CoV-2 and PEDV in 1) the supernatant immediately after the first centrifugation of the 10% homogenate, 2) the supernatant after overnight incubation at 4 °C and before concentration using the improved PEG precipitation method, 3) the supernatant after performing the improved PEG precipitation method and 4) the pellet. The spike test was performed on all three oyster lots and the average and standard error values were calculated.

### 2.4 Oyster samples for survey

**Figure 1:**
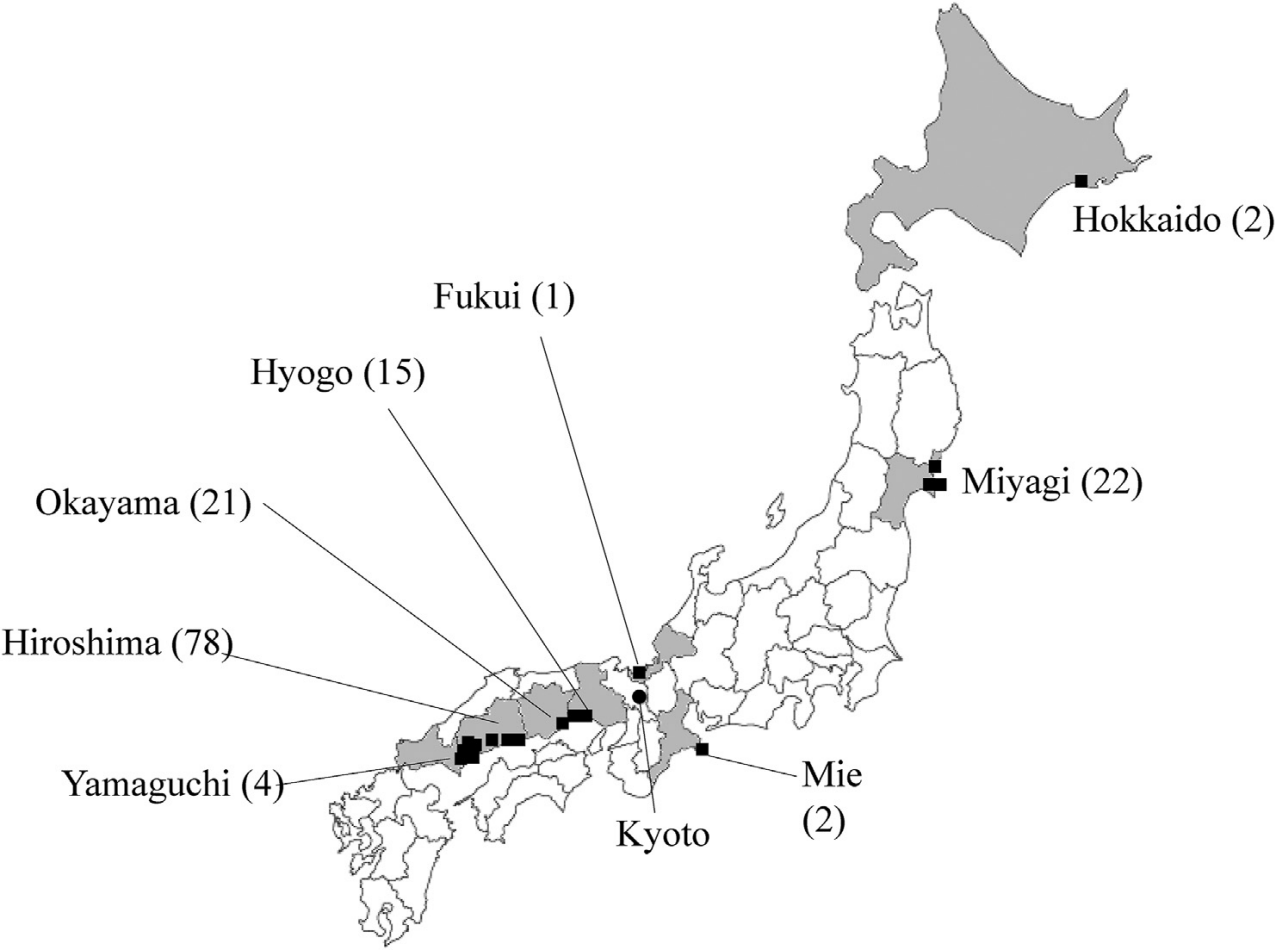
Map of Japan showing the locations of oyster-producing areas. Numbers in parentheses indicate the number of oyster samples we purchased from supermarkets in Kyoto that had been cultivated in each region.

We examined 145 cultivated oyster samples, consisting of 53 for raw consumption and 92 for cooked consumption, sold at 23 supermarkets in Kyoto from October 2021 to April 2022. The oysters were cultivated and processed in 31 coastal areas in nine prefectures throughout Japan, as the oysters sold in Kyoto supermarkets are purchased randomly. We obtained a vast number of oysters, 81.4% (118/145), from the Seto Inland Sea area (Hiroshima, Okayama, Hyogo and Yamaguchi prefectures), where oyster aquaculture is very popular (Figure 1). A total of 138 of the 145 oysters were packed with sterile seawater. The remaining seven were packed without seawater (referred as waterless packs: WLP), and water leakage was visible to the naked eye in the packs of these oysters; these samples were considered unfresh (S-Figure 1). Of these 145 samples, 92 were heat-eating oysters, which are shipped immediately after aquaculture is completed, and 53 were raw-eating oysters, which are circulated in sterile seawater with exposure to ultraviolet light before shipment after aquaculture is completed. Oyster samples were delivered to our laboratory in a refrigerated transport container and kept at 4 °C in the laboratory until use.

### 2.5 Preparation of 10% homogenates of midgut glands

For samples packed in seawater, virus concentration and removal of midgut-gland contaminants were performed using a combination of existing authorized PEG precipitation methods reported by the Ministry of Agriculture, Forestry and Fisheries (Imamura et al., 2021; MAFF, 2019), Ministry of Health, Labour and Welfare (MHLW, 2003) and National Institute of Health Sciences (NIHS, 2010) in Japan with minor modifications. Briefly, using sterile scissors and tweezers, the midgut gland was removed from 5 to 10 oysters in the same pack, with care taken to include as little of the surrounding white tissue as possible, and 3 g was transferred to a filtered stomacher bag. The remaining midgut-gland tissue was promptly stored at −80 °C in preparation for re-testing. After adding 9x volume of phosphate-buffered saline (PBS, 27 mL) to the filtered stomacher bag, solid pieces of tissue were crushed manually by repeated pressing an empty 50 mL tube over the top of the filtered stomacher bag for 30 s to make a 10% homogenate. Details of the homogenizing procedure are shown in the Supplemental Video files 1–4. The 10% homogenate was filtered through the filter portion of the stomacher bag, and a minimum of 18 mL was transferred to the non-filter portion of a new filtered stomacher bag using a whole pipette. After filtering a second time through the filter section of the new filtered stomacher bag, 12 mL of the 10% homogenate was transferred into a 15-mL tube using a whole pipette.

Supplementary video related to this article can be found at https://doi.org/10.1016/j.heliyon.2022.e10864

The following is/are the supplementary data related to this article:

S-Video-1-Removing-Midgut-Gland-from-Oyster S-Video-1-Removing-Midgut-Gland-from-Oyster

S-Video-2-Homogenizing-Manually-for-30-Seconds S-Video-2-Homogenizing-Manually-for-30-Seconds

S-Video-3-Filtration S-Video-3-Filtration

S-Video-4-Pippeting

### 2.6 Preparation of the whole process control

PEDV (NK94-P6) was used as the whole process control for the spike test. PEDV NK94-P6 supernatant from Vero cell cultures stored at −80 °C in 1.5- mL screw-capped tubes was thawed before diluting 1 μL with 99 μL of PBS to make a 1:100 solution of PEDV. Subsequently, 1 μL of this 1:100 solution was added to a 15-mL tube containing 12 mL of 10% homogenate (the final concentration of the spiked virus was 100,000 copies).

### 2.7 Virus concentration and removal of midgut-gland contaminants using a modified PEG precipitation method

After spiking PEDV, 30 mg of α-amylase (Wako Pure Chemicals Co., Ltd, Osaka, Japan) was added to the tube to digest organic substances derived from oysters. After vortexing, the tube was incubated at 37 °C for 1 h at 6 rpm. Subsequently, the tube was vortexed again and centrifuged at 8000 × g for 20 min at 4 °C.

A 9-mL volume of the PEDV supernatant prepared above was added to a new 15-mL tube, followed by PEG solution (3.6 mL), and the mixture was mixed by inversion and vortexing. After standing at 4 °C for 18–22 h to allow the virion to adsorb to PEG and midgut glands, the tubes were centrifuged at 8000 × g for 20 min at 4 °C without vortexing. The resulting supernatant was discarded except for approximately 150 μL at the bottom of the tube, which may be virion-enriched, along with the pellet derived from midgut-gland tissue. To further homogenate the pellet, which may contain adsorbed virion, and remove midgut-gland residue from the tube wall, 250 μL of PBS was added to the tube along with an empty 1-mL pipette tip. The mixture was then vortexed with the pipette tip still inside to help dislodge clumps of tissue adhering to the tube wall. Approximately 400 μL of the resulting homogenate was transferred to a 1.5-mL screw-capped tube for RNA extraction (referred to hereafter as 400-μL extraction). If the extraction efficacy of the PEDV-spiked sample was less than 1% (corresponding to a cycle threshold (C_t_) value above 35.909), the results were rejected, and we thawed and re-tested midgut-gland samples stored at −80 °C. Upon retest, we retained 400 μL, instead of 150 μL, of the supernatant generated after PEG precipitation. The final solution was then made up to 1 mL by adding 600 μL of PBS (referred to hereafter as 1-mL extraction).

For samples in WLP, three concentrations of homogenates (2%, 5%, and 10%) were prepared to account for the water leakage in the packs obtained from the supermarkets. To achieve the same concentration of PEDV per midgut-gland weight compared to the 10% homogenate, one-fifth and half of the volume of thawed PEDV were added to the 2% and 5% homogenates, respectively. Because we could not obtain sufficient amounts of midgut glands, we omitted the 400-μL extraction of RNA from 5% to 2% homogenates and only performed the 1-mL extraction. For samples with deficient amounts of midgut glands, we did not re-test 1 mL-extractions even if the real-time RT-PCR C_t_ value for PEDV showed less than 1% extraction efficacy for the 10% homogenate (Table 1). In all other cases, virus concentration and removal of midgut-gland contaminants were performed using the same protocol as that for oysters packed with seawater.

**Table 1:** Comparison of Ct values of real-time RT-PCR to detect spiked PEDV by homogenate concentration in waterless packed oysters.

Serial No.	Date of	Prefecture where oysters were farmed.	Homogenate Concentration			
	sampling		10%	2%	5%	10%
			400-ul extraction	1-ml extraction		
WLP-1	05/10/2021	Hokkaido	31.616	ND	ND	ND
WLP-2	11/10/2021	Hokkaido	30.371	ND	ND	ND

WLP-3	24/11/2021	Hiroshima	30.118	31.524	31.990	31.211
WLP-4	11/01/2022	Hiroshima	38.342	32.482	31.983	32.516
WLP-5	21/02/2022	Mie	43.579	32.299	32.278	ND
WLP-6	01/03/2022	Mie	37.130	33.020	32.837	34.005
WLP-7	22/03/2022	Mie	38.108	33.688	32.356	ND

Average between WLP-3 and WLP-7			37.455	32.603	32.289	32.577
SD			5.051	0.810	0.349	1.398
						

ND: Not Done

Note that the oysters in the waterless packs were packed in the on-site preparation facilities of supermarkets in Kyoto City, not in the farming prefecture.

### 2.8 Extraction of viral RNA and real-time RT-PCR

For both the 400-μl and 1-mL extraction, each homogenate was eluted into 50 μl of RNase-free water using the mag LEAD 6 g C automated extraction platform (Precision System Science Co., Matsudo, Japan) and reagent cartridges (MagDEA Dx SV, Precision System Science; MagDEA Dx MV II, Precision System Science) for immediate real-time RT-PCR analysis. Real-time RT-PCR was performed using a QuantStudio 3 (Thermo Fisher Scientific, Inc., Waltham, MA, U.S.A.), following an Emergency Use Authorization issued by the US Food and Drug Administration (FDA) (Lu et al., 2020) based on the N2 sequences for SARS-CoV-2 detection, and the report by Zhou et al. (2017) for PEDV detection. Detection of the two viruses was conducted simultaneously using the rapid protocol for SARS-CoV-2 detection in accordance with the Emergency Use Authorization issued by the FDA (Lu et al., 2020). For PEDV detection, primer and probe concentrations described by Zhou et al. (2017) were doubled as a safety measure. In addition, PrimeDirect® Probe RT-qPCR mix (TaKaRa Bio Inc., Otsu, Japan), which is resistant to potential PCR inhibitors, was used to minimize the PCR inhibiting effects of the midgut-gland. By the instructions for this reagent, we added a 95 °C for 30 s step before real-time RT-PCR. In brief, for SARS-CoV-2 detection, 50-μL real-time RT-PCR reactions comprised 25 μL of 2x PrimeDirect® Probe RT-qPCR Mix (TaKaRa Bio), 5 μL of primer-probe mix for SARS-CoV-2 N2 detection (Integrated DNA Technologies, Inc, Singapore), 0.5 μL of Rox II Dye, 9.5 μL of nuclease free water, and 10 μL of the RNA template. For PEDV detection, the 5-μL of primer-probe mix contained 6.7 μM of each primer (forward: 5′-CAGGACACATTCTTGGTGGTCTT-3′, reverse: 5′-CAAGCAATGTACCACTAAGGAGTGTT-3′) and 1.7 μM of probe (5′-FAM-ACGCGCTTCTCACTAC-MGB-3′), while the other reagents were unchanged. The cycling conditions were as follows for both viruses: one cycle at 95 °C for 30 s and 55 °C for 300 s, followed by 45 cycles each at 95 °C for 3 s, and 55 °C for 30 s. The automatically calculated C_t_ value was adopted and the C_t_ cut-off was set at 40.000.

### 2.9 Determining the limit of detection (LOD) and extraction efficacy

The following commercially available DNA sequence derived from the recombinant plasmid was used as a positive control: 2019-nCoV_N_Positive Control for SARS-CoV-2 (Integrated DNA Technologies). The positive control (200,000 copies/μL) was subjected to 10-fold serial dilutions up to 10^−7^ with purified water and used to generate a standard curve for real-time RT-PCR. The LOD, indicative of the analytical sensitivity of the real-time RT-PCR assay, was determined in duplicate analysis. For PEDV, the extraction efficacy was calculated according to the ISO (2017) and MAFF (2020) descriptions by adding the same amount of the NK94-P6 supernatant to the buffer as we added PEDV to oyster midgut-gland homogenates and using the value obtained as the reference value.

## 3 Results

**Figure 2:**
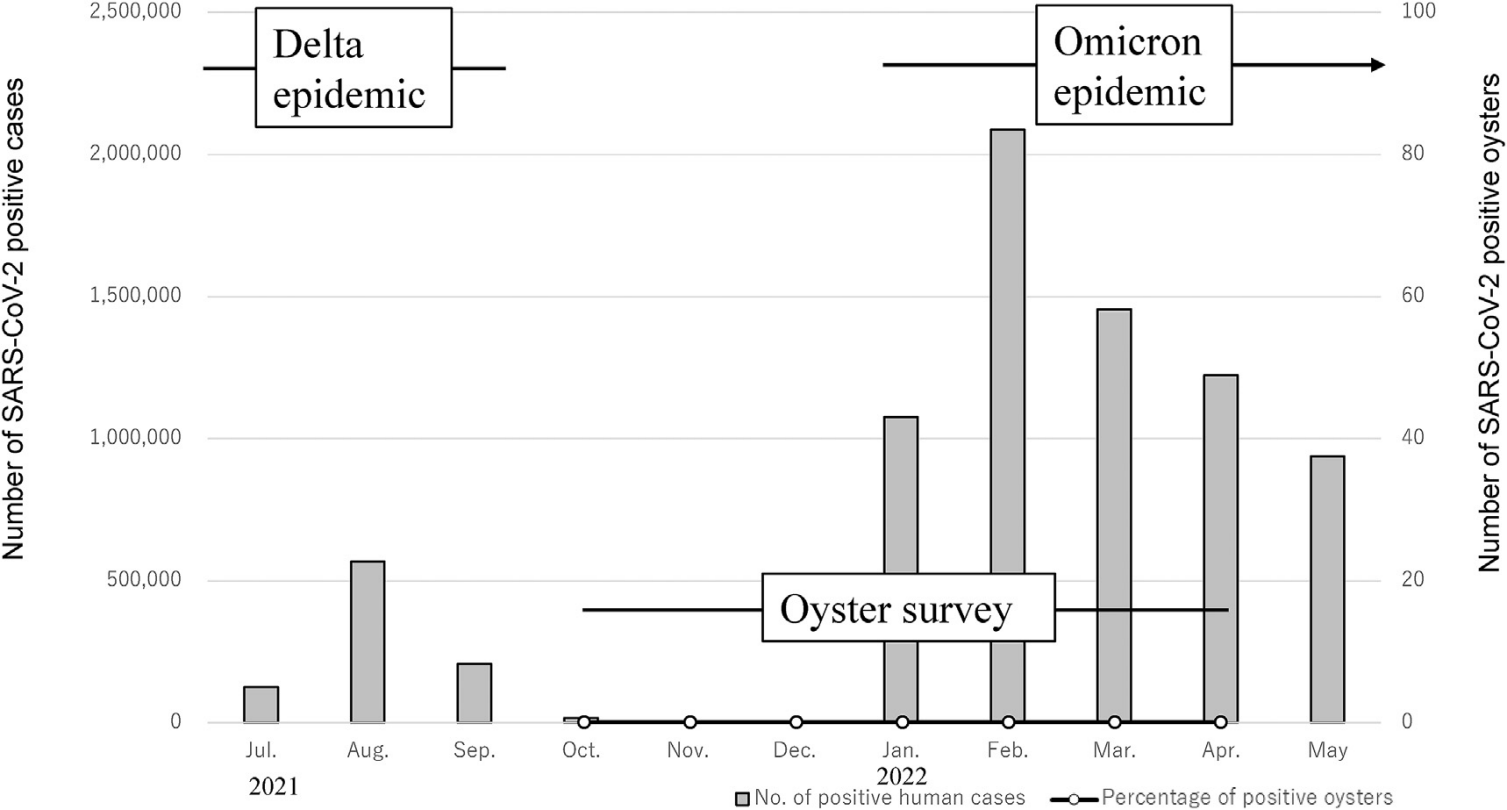
Number of positive cases of SARS-CoV-2 in Japan and the timing of the oyster survey in relation to the Delta and Omicron variant-related epidemics.

**Figure 3:**
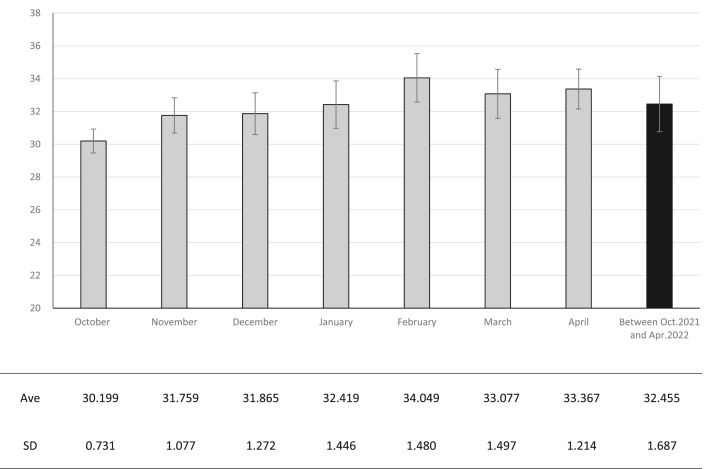
Monthly variation in real-time RT-PCR C_t_ values for PEDV in midgut glands of oysters packed in seawater.

**Figure 4:**
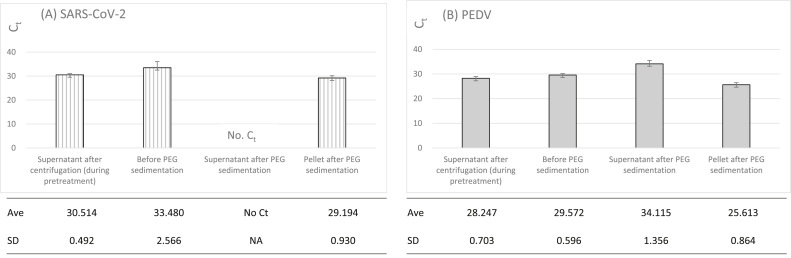
Real-time RT-PCR C_t_ values for SARS-CoV-2 (A) and PEDV (B) at various stages of the modified PEG precipitation method. Note: The concentration of PEDV used in the spike test was 100 times the amount of PEDV added to the 145 oyster samples.

Our investigation, which was conducted during the rise in Omicron variant related infections worldwide, including in Japan, found no SARS-CoV-2 in any of the 145 commercially purchased oysters, which were cultivated in nine prefectures and sold at supermarkets in Kyoto, regardless of region of origin or whether the oysters were for raw or cooked consumption (Figures 1 and 2, S-Table 1). In contrast, PEDV, used as whole process control, was detected in all 145 samples, as expected (Figure 3). A comparison of C_t_ values before and after the modified PEG precipitation method showed that values were reduced by 4.286 and 3.959 for samples spiked with SARS-CoV-2 and PEDV, respectively, confirming that the method was effective for enriching the virions of interest (Figure 4).

As shown in Table 1, Figure 3 and S-Table 1, C_t_ values indicating levels of PEDV in the 145 oyster samples varied depending on the month the oysters were purchased and whether they were packed with or without seawater. Specifically, smaller C_t_ values were obtained for the 5% and 2% homogenates than the 10% homogenates derived from oysters in WLP. Table 1 shows that while four of the seven samples from WLP had an extraction efficiency below 1%, with C_t_ values ranging from 37.130 to 43.579 in 10% homogenates, the C_t_ range decreased to 31.983 to 32.837 in 5% homogenates, and to 32.299 to 33.020 in 2% homogenates, indicating that decreasing the homogenate concentration raised the recovery rate to above 1%. No differences were observed between oysters meant for raw and cooked consumption (S-Table 1).

**Figure 5:**
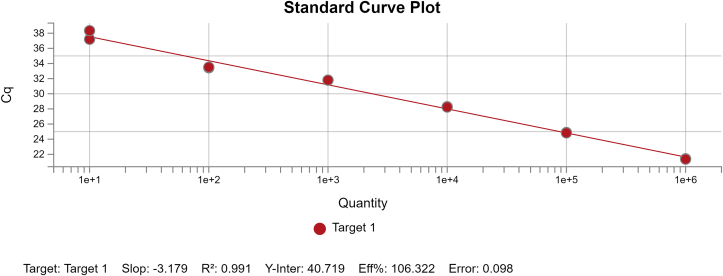
Standard curve of real-time RT-PCR for SARS-CoV-2.

Additionally, as shown in Figure 3, a comparison of C_t_ values obtained from 138 PEDV-spiked oyster samples that were packed in seawater from supermarkets showed that the mean monthly C_t_ value increased from 2.878 to 3.811 from February to April 2022 and from 1.484 to 2.020 from November to January 2021, compared to October. As shown in Figure 5, the LOD determined by real-time RT-PCR assay for SARS-CoV-2 was 1.0 × 10^1^ copies/reaction. Analysis of reproducibility revealed that there was a strong linear correlation (R^2^ > 0.990) between copy number and C_t_ value, meeting the accuracy control criteria set by ISO (2017): R^2^ > 0.991, slope = −3.179, y-intercept = 40.719, efficiency = 106.322%.

## 4 Discussion

We surveyed cultivated oysters for the presence of SARS-CoV-2 from October 2021 to April 2022, including the period in which the Omicron variant emerged worldwide, including in Japan. We did not detect SARS-CoV-2 in any of the 145 oysters sampled, which were cultivated in nine prefectures throughout Japan and purchased by supermarkets in Kyoto. Our finding indicates that consumption of oysters is an extremely unlikely risk factor for COVID-19 infection.

Bivalves, including cultivated oysters, are filter feeders known to bioaccumulate norovirus and cause gastroenteritis in humans when consumed undercooked (Hassard et al., 2017; Ueki et al., 2005). Tests conducted by Japan's MAFF on domestically cultivated oysters show that the norovirus-positive rate ranges from 19% in October to 90% in March, with an average rate of 46% for an entire year (MAFF, 2020). Thus, we conducted the present study out of concern that SARS-CoV-2 may pose a similar risk. However, our study revealed that the risk of COVID-19 infection via consumption of oysters is extremely low.

Several studies have reported detecting SARS-CoV-2 in sewage collected from various facilities in Japan and other countries (Ali et al., 2021; Jones et al., 2020; Kitajima et al., 2020; Hata et al., 2021; Tran et al., 2021). This study aimed to understand the behavior of SARS-CoV-2 in sewage systems to determine whether it could be a new source of human infection. Noroviruses, like SARS-CoV-2, have also been detected in sewage. According to several reports, noroviruses shed from humans bioaccumulate in cultivated oysters, the consumption of which then forms a source of transmission to humans (Hassard et al., 2017; Ueki et al., 2005). After multiplying in the intestinal tract of infected individuals, noroviruses are released into the sewage system through toilets and finally into rivers and oceans after being chlorinated in sewage treatment plants (Hassard et al., 2017; Randazzo et al., 2018). As noroviruses are resistant to a variety of disinfectants, they remain infectious in the environment for an extremely long period (Hassard et al., 2017; Randazzo et al., 2018). Therefore, once shed in human feces, complete elimination from the environment is impossible and the virus continues to circulate.

Wastewater monitoring has been attempted in Japan for early detection of COVID-19 outbreaks in nursing homes and other facilities (Kitajima et al., 2020; Hata et al., 2021). However, this is done in limited areas and is not linked to the location of oyster aquaculture environments. In other words, comprehensive SARS-CoV-2 testing for the aquatic environment is not done in Japan because it is also a sensitive issue. Therefore, the dynamics of SARS-CoV-2 in the aquatic environment in the production areas of 118 oysters (Hiroshima, Okayama, Hyogo, and Yamaguchi prefectures in the Seto Inland Sea), representing 81.4% of the 145 oysters we purchased in Kyoto city, is unknown. Norovirus, which is shed into the aquatic environment from human feces and SARS-COV-2, is frequently detected in cultivated oysters throughout Japan, including the Seto Inland Sea (MAFF, 2021, 2022). There was no significant difference in the detection rate of norovirus between the presence or absence of virus purification treatments such as filter filtration and UV irradiation for raw-eating consumption (MAFF, 2021, 2022). Therefore, it can be inferred that the aquatic environment in this area may be contaminated with various viruses of human fecal origin such as norovirus and SARS-COV-2.

In contrast, SARS-CoV-2 easily loses its infectivity and is extremely susceptible to some disinfectants, including chlorine, 0.5% sodium hypochlorite, and 70% ethanol (Jones et al., 2020; Tran et al., 2021). Further, according to previous findings, the bioaccumulation efficiency of SARS-CoV-2 in oysters is lower than that of non-enveloped viruses (Brnić et al., 2022; Desdouits et al., 2021; Jones et al., 2020). Therefore, we predict that a key reason why SARS-CoV-2 could not be detected in commercially cultured oysters in Japan may be because of its low viability in the environment. In our study, 53 raw-eating oysters, which were circulated in sterile seawater with exposure to ultraviolet light before shipment after aquaculture is completed, were all negative for SARS-CoV-2, and even 92 heat-eating oysters that were shipped immediately after the end of aquaculture were all negative for SARS-CoV-2. Furthermore, the fact that no cases of COVID-19 infection from eating oysters have been reported worldwide may also reinforce this prediction.

One limitation of this study is that the heat-inactivated SARS-CoV-2 used to spike oyster samples showed a reduction in C_t_ value of just 1.320 using the modified PEG precipitation method (Figure 4). In contrast, a 2.634 reduction was achieved for infectious PEDV obtained from Vero cell culture supernatants, which was measured simultaneously. It is tempting to speculate that the difference in performance between the two coronaviruses is due to the fact that heating partially disrupts the viral surface of SARS-CoV-2, while PEDV retains a robust viral coating and its infectiousness. However, the former condition is likely to be closer to reality than the latter since it is likely that SARS-CoV-2 exists in the environment in a damaged form. Nevertheless, the results of the SARS-CoV-2 test conducted on commercial oysters in this study should be interpreted with caution since the presence of trace amounts of SARS-CoV-2 below the detection sensitivity of our instruments may have caused false-negative results.

In addition, as shown in Figure 3, we observed a trend toward higher C_t_ values using our whole process control for oyster samples obtained throughout the study period from October 2021 to April 2022, but especially from February to April. We noticed more white tissue, presumed to be fat and/or glycogen components, adhering to the peri midgut-gland area on gross examination, especially in samples obtained from February to April. In samples obtained from February to April 2022, we also observed a particularly large amount of solids visible to the naked eye in the supernatant of the 10% homogenate, especially on the lid of the 15-mL tube and on the tube wall. Furthermore, as shown in Table 1 and S-Figure 1, C_t_ values were higher in oysters that had spontaneously leaked water in their packages from the supermarket. Decreasing the homogenate concentration from 10% to 5% or 2% and using a 1-mL volume resulted in lower C_t_ values than those obtained for the 400-μL extraction of the 10% homogenate.

It should be noted that oysters store nutrients, especially during the winter months, in preparation for spring spawning, which can lead to changes in composition within the oyster body that may negatively affect laboratory test results. Additionally, the condition of the oysters upon receipt can also cause artificial effects. For example, oysters leaking water from their bodies can have concentrated midgut-gland components, leading to excessive amounts of inhibitors of PEG precipitation and/or PCR amplification in the sample. Separately to our study, a validation of the ISO method using mussels reported an increase in C_t_ values due to inhibitory effects in certain lots (Lowther et al., 2019). Hence, as a safeguard, a 5% homogenate, which can buffer out excess inhibitory effects against PEG precipitation, and a 1-mL extraction, which allows for stronger digestion and removal of inhibitory substances, may provide a more accurate survey.

Previous studies on the detection of norovirus in the oyster midgut gland have, after PEG precipitation, further centrifuged the oyster sample to remove foreign substances derived from the midgut gland that can inhibit gene amplification, and removed fragmented RNAs in the midgut gland that can potentially lead to overestimation of the infection risk to humans, and used the resulting supernatant for RNA extraction (Imamura et al., 2021, MAFF, 2019). However, some researchers have pointed out that the final centrifugation step causes SARS-CoV-2 and noroviruses to adsorb nonspecifically to the pellet containing foreign substances, reducing the virus extraction efficacy (Brnić et al., 2022; Miura et al., 2018; Yamazaki, personal communication). Given the variety of substances that inhibit PCR amplification, it can be difficult to identify the ones affecting an individual setup (Schrader et al., 2012; Wilson, 1997). A previous study using a common method adopted in Japan for detecting norovirus in oysters (MAFF, 2019) reported that 188 (36.9%) of 510 fresh oyster samples purchased directly from producers had a recovery rate of less than 1% (Imamura et al., 2021). The findings from this previous study infer that substances derived from the midgut gland can inhibit viral enrichment by PEG precipitation, real-time RT-PCR amplification efficiency, or both.

To reduce the inhibitory effects of oyster-derived substances, we examined the conditions that would allow for their comprehensive digestion and removal, including from midgut glands. Therefore, in our modified PEG precipitation method, we used α-amylase to digest glycogen derived from oysters and extracted the pellet and PEG precipitate simultaneously without separation with a final centrifugation step. Furthermore, we used PrimeDirect® Probe RT-qPCR Mix, an enzyme resistant to real-time RT-PCR inhibitors, for more stable detection. Using these strategies, we obtained an extraction efficacy of 1% or higher in 124 of the 138 (89.9%) samples packed with seawater on the first try using 400-μL extraction. Upon retesting of the remaining 14 samples (10.1%) using 1-mL extraction, 11 samples showed extraction efficiencies greater than 1%, while the remaining three samples continued to show extraction efficiencies less than 1%. Therefore, 135 samples (97.8%) showed an extraction efficiency of more than 1% (S-Table 1). A previous study by Brnić et al. reported that the control extraction efficiency of 96.1% (74/77) of tested bivalve mollusks met the established criterion (extraction efficiency >1%), showing the same level of accuracy as that in our present study, which specifically detected infectious virions. Furthermore, our concept of extracting RNA from all pellets containing foreign material and both concentrated and non-specifically adsorbed viral particles after PEG precipitation can be used to improve the accuracy of detecting viruses in wastewater sewage, which suffers from low recovery rates due to non-specific adsorption of viruses (Brnić et al., 2022; Miura et al., 2018).

As shown in Figure 3, the oyster sample obtained in October, which had the fewest contaminants visible to the naked eye, showed the lowest mean C_t_ value of 30.199 and, thus the highest virus extraction efficacy. Given the increase in potential substances that can inhibit PEG sedimentation or real-time PCR amplification related to the abovementioned seasonal changes in the properties of the oyster body, we observed a slight decrease in recovery from November to January and a mild decrease from February to April. However, these represented only 3.0- to 4.7-fold decreases from November to January and 7.4- to 14.4-fold decreases from February to April, suggesting that this simplified method can maintain high viral extraction efficacy. Furthermore, if we can anticipate the inhibitory effects related to the concentration of inhibitors in the oyster body due to water leakage shown in Table 1 and S-Figure 1, it may be possible to improve the virus extraction efficacy by decreasing the midgut-gland homogenate from 10% to 5% and using a larger volume (1 mL rather than 400 μL) for RNA extraction.

Japan’s sewage treatment penetration rate was 80.1% as of 2020, which we assume has contributed to preventing the spread of SARS-CoV-2 in the environment and its accumulation in cultivated bivalve mollusks. In contrast, the risk of COVID-19 infection from consumption of cultivated bivalves may be much higher in countries with low sewage treatment coverage and a greater number of patients. Ingestion of bivalves in countries with inadequate sewage treatment or high patient populations, where trace amounts of SARS-CoV-2 have been detected in non-cultivated bivalves exposed to untreated sewage water in the natural environment and in cultivated bivalve mollusks in oyster farms (Mancusi et al., 2022; Polo et al., 2021), may further increase the risk of COVID-19 infection. However, the results of a survey using the modified method developed in this study, which removes virus RNA fragments and focuses on the detection of virions, suggest that the risk of SARS-COV-2 infection via consumption of commercial oysters is extremely low in Japan.

## Declarations

### Author contribution statement

Yasuko Yamazaki; Wataru Yamazaki: Conceived and designed the experiments; Performed the experiments; Analyzed and interpreted the data; Wrote the paper.

Uraiwan Thongchankaew-Seo: Conceived and designed the experiments; Performed the experiments; Analyzed and interpreted the data.

### Funding statement

This work was supported by the Ministry of Education, Culture, Sports, Science and Technology (MEXT) and Japan Society for the Promotion of Science, Grants-in-Aid for Scientific Research - JSPS KAKENHI (grant number JP21H03180).

### Data availability statement

Data included in article/supp. material/referenced in article.

### Declaration of interests statement

The authors declare no conflict of interest.

### Additional information

No additional information is available for this paper.

## Appendix A Supplementary data

S-Figures-Yamazaki-Heliyon_V2 S-Figures-Yamazaki-Heliyon_V2

S-Table1-Yamazaki-Food-Control S-Table1-Yamazaki-Food-Control
