# Supplementary material for: Very low likelihood that cultivated oysters are a vehicle for SARS-CoV-2: 2021–2022 seasonal survey at supermarkets in Kyoto, Japan
Source: Heliyon. 2022 Oct 6;8(10):e10864. doi: 10.1016/j.heliyon.2022.e10864 (PMC9535880; doi:10.1016/j.heliyon.2022.e10864)
Supplement: S-Table1-Yamazaki-Food-Control [file mmc2.pptx]

## Slide 1
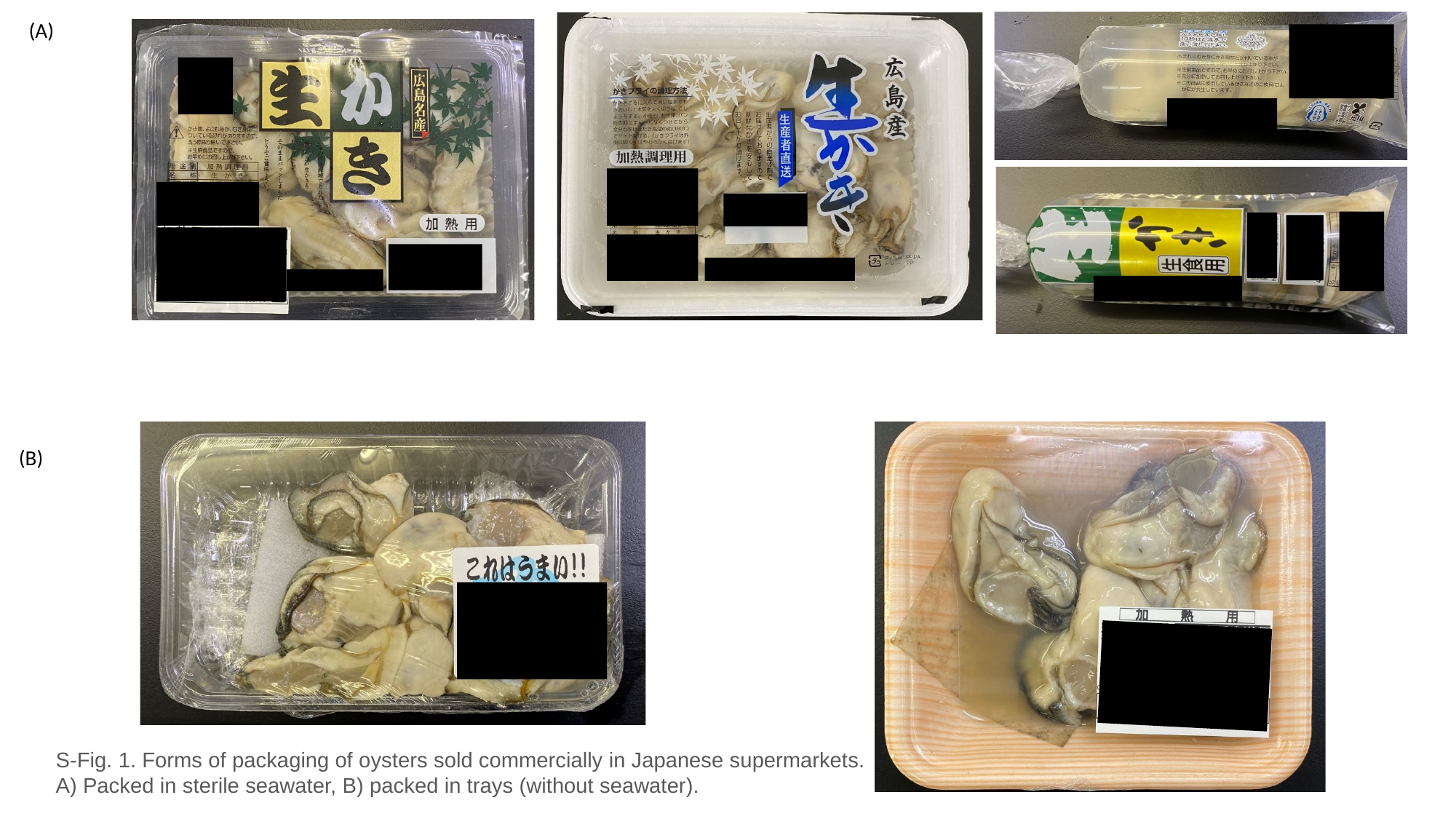

(A)
(B)
S-Fig. 1. Forms of packaging of oysters sold commercially in Japanese supermarkets.
A) Packed in sterile seawater, B) packed in trays (without seawater).
